# Molecular typing of *Staphylococcus aureus* based on coagulase gene

**DOI:** 10.14202/vetworld.2018.423-430

**Published:** 2018-04-06

**Authors:** Faizan Javid, Anil Taku, Mohd Altaf Bhat, Gulzar Ahmad Badroo, Mir Mudasir, Tanveer Ahmad Sofi

**Affiliations:** 1 Bacteriology Laboratory, Division of Veterinary Microbiology and Immunology, Sher-e-Kashmir University of Agricultural Sciences and Technology of Jammu, Jammu and Kashmir, India; 2 Division of Veterinary Pathology, Sher-e-Kashmir University of Agricultural Sciences and Technology of Jammu, Jammu and Kashmir, India; 3 Division of Animal Genetics and Breeding, Sher-e-Kashmir University of Agricultural Sciences and Technology of Jammu, Jammu and Kashmir, India

**Keywords:** coagulase, restriction fragment length polymorphism, sequence-based phylogenetic analysis, *Staphylococcus aureus*

## Abstract

**Aim:**

This study was conducted to study the coagulase gene-based genetic diversity of *Staphylococcus aureus*, isolated from different samples of cattle using restriction fragment length polymorphism (RFLP) and their sequence-based phylogenetic analysis.

**Materials and Methods:**

A total of 192 different samples from mastitic milk, nasal cavity, and pus from skin wounds of cattle from Military Dairy Farm, Jammu, India, were screened for the presence of *S. aureus*. The presumptive isolates were confirmed by *nuc* gene-based polymerase chain reaction (PCR). The confirmed *S. aureus* isolates were subjected to coagulase (*coa*) gene PCR. Different *coa* genotypes observed were subjected to RFLP using restriction enzymes *Hae111* and *Alu1*, to obtain the different restriction patterns. One isolate from each restriction pattern was sequenced. These sequences were aligned for maximum homology using the Bioedit softwareandsimilarity in the sequences was inferred with the help of sequence identity matrix.

**Results:**

Of 192 different samples,39 (20.31%) isolates of *S. aureus* were confirmed by targeting *nuc* gene using PCR. Of 39 *S. aureus* isolates, 25 (64.10%) isolates carried *coa* gene. Four different genotypes of *coa* gene, i.e., 514 bp, 595 bp, 757 bp, and 802 bp were obtained. Two *coa* genotypes, 595 bp (15 isolates) and 802 bp (4 isolates), were observed in mastitic milk. 514 bp (2 isolates) and 757 bp (4 isolates) *coa* genotypes were observed from nasal cavity and pus from skin wounds, respectively. On RFLP using both restriction enzymes, four different restriction patterns P1, P2, P3, and P4 were observed. On sequencing, four different sequences having unique restriction patterns were obtained. The most identical sequences with the value of 0.810 were found between isolate *S. aureus* 514 (nasal cavity) and *S. aureus* 595 (mastitic milk), and thus, they are most closely related. While as the most distant sequences with the value of 0.483 were found between *S. aureus* 514 and *S. aureus* 802 isolates.

**Conclusion:**

The study, being localized to only one farm, yielded different RFLP patterns as observed from different sampling sites, which indicates that different *S*. *aureus* coagulase typeshave a site-specific predilection. Two *coa* patterns were observed in mastitic milk indicating multiple origins of infection, with 595 bp *coa* genotype being predominant in mastitic milk. The *coa* genotypes and their restriction patterns observed in the present study are novel, not published earlier. 514 and 595 *coa* variants of *S. aureus* are genetically most related.

## Introduction

*Staphylococcus aureus* is considered as a significant pathogen of animals and humans. It is regarded as the most common and prominent causative agent in all forms of mastitis. It is the major prevalent organism causing subclinical mastitis in India [1]. Economic losses to dairy industry due to *S. aureus* result from subclinical mastitis accompanied by a reduction in the quality and quantity of milk production [2]. *S. aureus* is also associated with pyogenic infections. *Staphylococcus* species are identified by their biochemical profile, colonial appearance, and hemolytic pattern. Biochemically, they are catalase positive and oxidase negative and utilize maltose. *S. aureus* produces different types of virulence factors such as capsules and adhesins, enzymes such as coagulase, catalase, hyaluronidase, and staphylokinase, and toxins such as α toxin, β toxin, δ toxin, leukocidin, enterotoxin, exfoliative toxin, and toxic shock syndrome toxin, which cause several disease conditions in humans and animals.

Coagulase is a major virulent factor, which is an enzyme secreted by *S. aureus* causing the clotting of plasma in the host. It causes conversion of fibrinogen to fibrin and fibrin production may shield *Staphylococcus* from phagocytosis. Species are classified into two types, coagulase-positive (CoPS) and coagulase-negative *S. aureus*. Clinical microbiologists consider coagulase production by *S. aureus* as important criteria for identification of *S. aureus*. *S. aureus* is characterized as coagulase-positive staphylococci and known as the main pathogen of mastitis infections in dairy animals [3]. Although the coagulase tube test is the standard phenotypic routine test used to identify *S. aureus* in biological samples, several groups have implemented the molecular analysis of the coagulase gene as an accurate defined test. The *coa* gene encoding coagulase protein is highly polymorphic because of the variable sequences (81 bp tandem repeats) at its 3’ end, which allows differentiation of *S. aureus* species [4]. This *coa* gene polymorphism is utilized as an epidemiological marker and typing is performed with primers homologous to a conserved region within the *coa* gene [5]. Since the number of repetitive sequences varies within the *coa* gene, the resulting polymerase chain reaction (PCR) products of individual strains can be of different lengths.

Molecular typing of *S. aureus* can be done based on different typing techniques. In the past decade, numerous molecular techniques have been developed and used for the identification and comparison of *S. aureus* isolates in epidemiological studies. The conventional typing procedures have several drawbacks; for example, phage typing as well as genotyping methods using pulsed-field gel electrophoresis are labor-intensive and time-consuming and can be performed only in specialized laboratories. Identification based on PCR-restriction fragment length polymorphism (RFLP) of the *coa* gene has been considered a more simple and accurate typing method for epidemiological investigations of bovine mastitis [6]. PCR-RFLP is a rapid, reproducible, simple, and efficient method for typing *S. aureus* isolated from a clinical specimen of human and animal sources. This typing technique helps in establishing genetic relations (lineages) between isolates from different sources [7]. PCR products (amplicons) are digested with the restriction enzymes *AluI* [8] or *HaeIII*. DNA fragments are separated by gel electrophoresis which can be compared by their RFLP. PCR products of different strains showing different RFLP patterns can be sequenced for both the strands of *coa* gene. The sequences can be aligned, and evolutionary lineages (phylogeny) based on the pair-wise distance between strains can be inferred.

This study aimed to study the coagulase gene-based genetic diversity of *Staphylococcus aureus*, isolated from different samples of cattle using restriction fragment length polymorphism (RFLP) and their sequence-based phylogenetic analysis.

## Materials and Methods

### Ethical approval

Ethical approval is not needed to pursue this type of the study. However samples were collected as per standard sample collection procedure without harming the animals.

### Samples

A total of 192 samples were collected aseptically from cattle of different age groups from Military dairy farm, Belicharana, Jammu, in a study period from October 2015 to May 2016. Of total 192 samples, 98 were mastitic milk samples (from clinical as well as subclinical mastitis), 44 pus samples from open wounds on skin, and 50 nasal samples. The samples were immediately taken to laboratory and stored at 4°C until further processing.

### Cultural identification of S. aureus

Mastitic milk samples were enriched in peptone water (PW) enrichment broth (HiMedia Pvt. Ltd., India). A loop full of milk sample was homogenized with sterile enrichment broth PW and incubated for 24 h at 37°C. The selective medium used for the isolation of *S. aureus* from mastitic milk was Baird Parker Agar (BPA) (HiMedia Pvt. Ltd., India). A loop full of inoculum from enrichment broth was streaked on BPA and incubated for 48 h at 37°C. The characteristic appearance of jet black colonies surrounded by a white halo was considered to be presumptive *S. aureus*. However, pus and nasal swab samples were streaked directly on selective medium Mannitol Salt Agar (MSA). The appearance of golden-yellow colonies was presumed to be *S. aureus*.

### Phenotypic detection of S. aureus

Phenotypic identification was done by demonstration of the typical cellular morphology in Gram’s stained smears, and Gram-positive cocci that occurred in clusters under the microscope were subjected to preliminary biochemical tests.

### Catalase test

A loopful of the young culture of bacterial isolates was mixed with a drop of 3% hydrogen peroxide over a clean glass slide. The production of gas bubbles or any effervescence within a few seconds was considered as catalase positive.

### Oxidase test

A loopful of test bacterial culture was placed on oxidase disc. The appearance of dark purple color within few seconds was noted as oxidase positive.

### Deoxyribonuclease (DNase) activity

For DNase test, appropriate quantity of DNase Test Agar with toluidine blue (HiMedia Pvt. Ltd., India) was dissolved in the required volume of distilled water and sterilized by autoclaving at 121°C for 15 min and poured in sterile Petri dishes. This medium was used for detecting the DNase activity of the bacteria after inoculating the plate with test organism. DNase produced by organism polymerizes DNA present in the media. This results in formation of a clear zone around the microbial growth which is visualized by flooding the plate with HCl.

### Tube coagulase test

Tube coagulase test was performed by placing 0.5 ml of rabbit plasma (HiMedia Pvt. Ltd., India) placed in small (7 mm) test tube. Two drops of an overnight broth culture of the *S. aureus* were added. The tube was rotated gently to mix the contents and then incubated at 37°C. The positive test was confirmed by clotting of plasma within 2-4 h.

### Extraction of bacterial DNA

Suspensions of isolated colonies of phenotypically identified *S. aureus*, isolated from all the three sampling sites, subculturedon nutrient agar (NA) slant were prepared in 1.5 ml microcentrifuge tubes in 250 µl of sterile double-distilled water by gentle mixing. The samples were boiled for 10 min, cooled on ice for 10 min, and centrifuged at 10,000× *g* in a table-top microcentrifuge (Biofuge Stratos, Heraeus) for 5 min. 2 µL of the supernatant was used as the template for PCR.

### Molecular detection of nuc gene of S. aureus isolates

The *S. aureus* isolates were confirmed by PCR using species-specific *thermonuclease* (*nuc)* gene (270 bp) primers *nuc*-F: GCGATTGATGGTGATACGGTT and *nuc*-R: AGCCAAGCCTTGACGAACTAAAGC [9]. The PCR amplification for *nuc* gene was performed in 25 µl reaction volume in 0.2 ml thin-walled PCR tubes using Mastercycler Gradient (Eppendorf, Hamburg, Germany). The reaction consisted of 2.0 µl template DNA, 5 µl of 5X buffer, 0.5 µl of 10 mM dNTP mix, 2.5 µl of 25 mM Mgcl2, 2 U of Taq DNA polymerase (Promega, USA), 0.5 µM concentration of each primer, and sterile nuclease-free water (Promega). The PCR conditions in Mastercycler Gradient Thermal cycler (Eppendorf, Germany) consisted of initial denaturation at 94°C for 5 min, followed by 35 cycles of 94°C for 1 min, 55°C for 30 s, and 72°C for 1 min. This was followed by final extension at 72°C for 7 min.

### Electrophoresis and documentation

Agarose gel (1% w/v) was made by heating the appropriate amount of agarose (Sigma-Aldrich, St. Louis, USA) with 30 ml 1X Tris-acetate EDTA (TAE) buffer in a 500 ml Erlenmeyer flask. The flask was cooled to 60°C, and ethidium bromide added to the final concentration of 0.5 µg/ml. The warm agarose solution was poured into a plastic holder with suitable comb (with 0.5-mm or 1-mm wells) and allowed to completely set at room temperature for 30 min. DNA samples were mixed with 6X loading dye and loaded in separate wells on the submerged gel. Standard molecular weight marker, 100 bp (Promega, USA), was also loaded in one well. The voltage 1-5 V/cm was applied across the gel unit tracking dye (Bromophenol blue) migrated to appropriate distance. The gel was removed and DNA bands were visualised under ultraviolet illumination and photographed with Gel Documentation System (BioDocAnalyze, Biometra, Germany). The molecular sizes of the DNA bands were analyzed in relation to molecular weight marker.

### Molecular detection of coagulase (coa) gene

The primers used for amplification of *coa* gene of *S. aureus* are *coa* F: ATAGAGATGCTGGTACAGG and *coa* R: GCTTCCGATTGTTCGATGC [10]. The PCR amplification was performed in 25 µl in 0.2 ml thin-walled PCR tubes (Eppendorf, Germany). The PCR mixture contained a final concentration of 2.5 µl of 5X coloured buffer, 2.5 µl of 25 mM MgCl_2_, 0.25 µl 25 µM concentration of each primer (Table-1), 0.5 mM concentrations of each 2’-deoxynucleoside 5’-triphosphate, and 2 U of Taq DNA polymerase (Promega, USA). The amplification cycles in Mastercycler Gradient Thermal cycler (Eppendorf, Germany) consisted of 94°C for 45 s, followed by 30 cycles of 94°C for 20 s, 57°C for 15 s, and 70°C for 15 s and final extension at 72°C for 2 min.

**Table-1:** *S. aureus* isolates obtained from different samples using *nuc* gene PCR.

Samples	Source	Number of samples	*S. aureus* isolates (*nuc* positive) (%)
Mastitic milk	Cattle	98	22 (22.4)
Nasal swabs	Cattle	50	8 (16)
Pus from wounds	Cattle	44	9 (20.4)
Total	Cattle	192	39 (20.31)

*S. aureus=Staphylococcus aureus*, PCR=Polymerase chain reaction

### RFLP of coagene product of S. aureus

Variation in restriction patterns of *coa* variable *S. aureus* isolates was determined by PCR-RFLP using *HaeIII* and *AluI* restriction endonucleases (Promega, U.S.A). For digestion with *AluI* restriction endonuclease, 10 µl of *coa* PCR product, 18 µl of nuclease free water, 2 µl of 10X Tango buffer, and 2 unit of *Alu*I enzyme were added, mixed gently in a 0.2 ml PCR tube, and incubated at 37°C for 2 h in a water bath. Similarly, for digestion with *HaeIII*, 2 µl of 10*Buffer, 2 units of *HaeIII* enzyme, 10 µl of *coa* PCR product, and 18 µl of nuclease-free water were added and then mixed gently in a 0.2 ml PCR tube and incubated in the same fashion as during *AluI* digestion.After 2 h of incubation, the digested products were run in 2.75% agarose gel prepared in 1X TAE buffer containing 0.5 µg/ml of EtBr. The digested products were electrophoresed for 1 h at 100 V and then visualized and documented under Gel Doc System (BioDocAnalyze, Biometra, Germany).

## Results

### Culture findings

**Figure-1:**
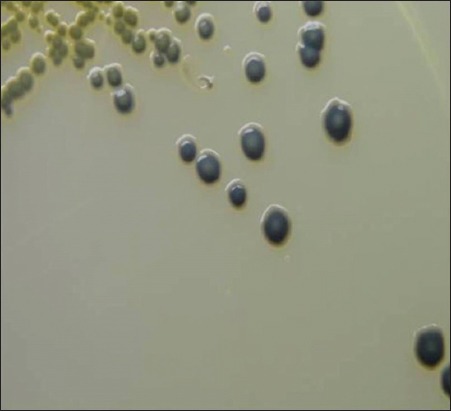
Typical jet-black colonies of *Staphylococcus aureus* surrounded by white halo showing lecithinase activity on Baird Parker Agar.

**Figure-2:**
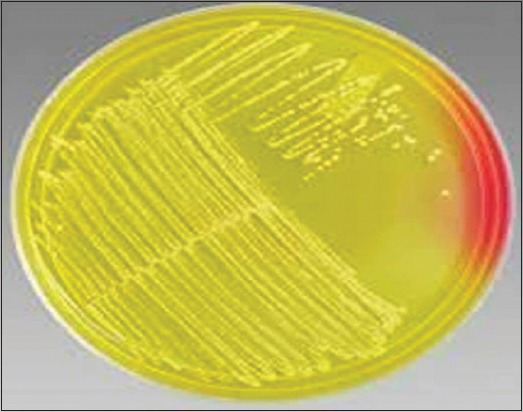
Typical golden-yellow colonies of *Staphylococcus aureus* on Mannitol Salt Agar.

Of total 98 mastitic milk samples, 84 samples (85.7%) revealed jet-black colonies surrounded by white halo indicating lecithinase activity on BPA medium, characteristic of *S. aureus*, as shown in Figure-1. Of 94 pus and nasal samples, 78 samples (82.9%) showed characteristic golden-yellow colonies of *S. aureus* (Figure-2). One pure well-isolated colony from all the BPA and MSA plates presumed to be *S. aureus* was subcultured on NA slant, to obtain 162 presumptive *S. aureus* cultures.

### Biochemical characterization

**Figure-3:**
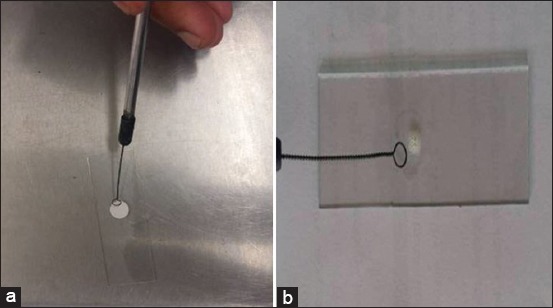
(a) Negative oxidase test. (b) Positive catalase test.

**Figure-4:**
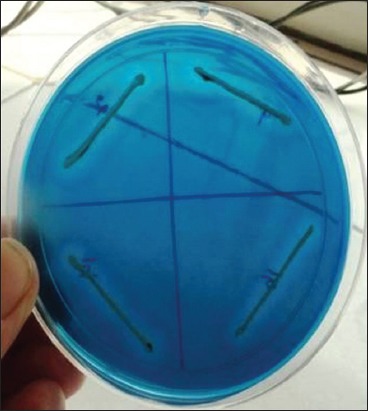
*Staphylococcus aureus* showing DNase activity on DNase agar.

**Figure-5:**
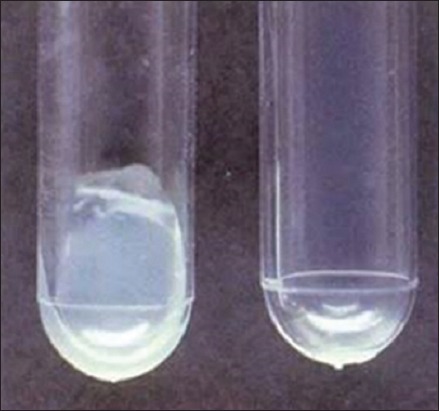
Coagulase positive (Clot), Coagulase negative (No Clot).

Subsequently, the colonies from NA slant were subjected to gram staining for morphological characterization and bacteria were typically Gram-positive cocci occurring in bunched, grape-like irregular clusters, which are characteristic of *Staphylococcus* spp. These *Staphylococcus* cultures were characterized biochemically for catalase and oxidase activity. Of 162 presumptive cultures, 114 (70.37%) cultures revealed positive catalase and negative oxidase activity (Figure-3a and b). Of 114 cultures, 75 (65.7%) cultures were positive for DNase test and showed typical depolymerization of DNA around the bacterial colonies on DNase agar as shown in Figure-4. Among the 114 cultures, only 22 (28.07%) cultures showed a positive result for tube coagulase test observed by the formation of the clot as shown in Figure-5.

### Molecular confirmation of S. aureus

**Figure-6:**
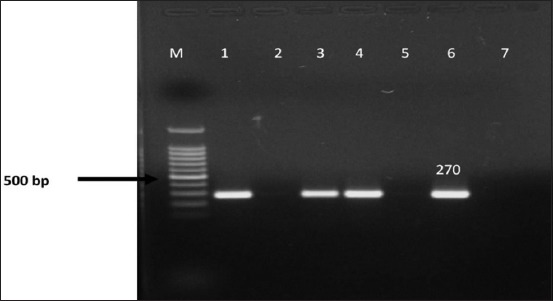
Polymerase chain reaction amplification of species-specific *nuc* gene of presumptive *Staphylococcus aureus* isolates. Lane M - 100 bp DNA ladder. Lane 1,3,4 - positive isolates. Lane 2 - negative isolate. Lane 5 - negative control. Lane 6 - positive control.

To validate our identification of *S. aureus*, all 114 cultures were subjected to genotypic characterization by PCR using species-specific *nuc* gene amplification. 39 (34.2%) cultures which were morphologically and biochemically identified as *S. aureus* revealed the presence of *nuc* gene by the amplification of 270 bp product of species-specific *nuc* gene (Figure-6 and Table-1).

### Molecular detection of coagulase gene (coa)

**Figure-7:**
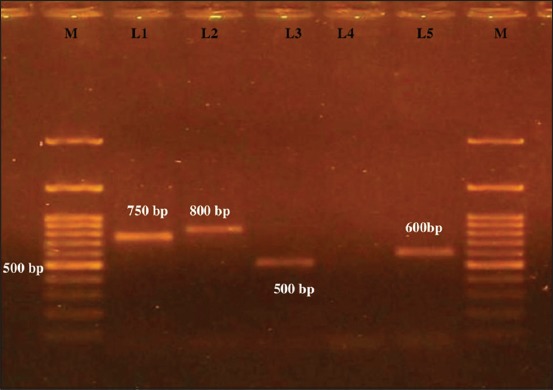
Polymerase chain reaction amplification of coagulase gene (coa) of Staphylococcus aureus isolates. M - 100 bp DNA ladder. L1 - 750 bp (approximately). L2 - 800 bp (approximately). L3 - 500 bp (approximately). L4 - negative control. L5 - 600 bp (approximately).

Twenty-five (64.10%) of 39 *S. aureus* isolates were positive for *coa* gene and four different amplicons of *coa* gene were detected (Table-2 and Figure-7). 22 isolates among them were positive for tube coagulase test.

**Table-2:** *coa* gene PCR amplification showing polymorphism ranging from 500 to 800 bp.

Samples	Source	*S. aureus* isolates	*coa* positive *S. aureus* (%)	*Coa* gene amplicon (bp)
Mastitic milk	Cattle	22	19 (86.3)	595 (15 isolates) 802 (4 isolates)
Nasal swabs	Cattle	8	2 (25)	514 (2 isolates)
Pus	Cattle	9	4 (44.4)	757 (4 isolates)
Total	Cattle	39	25 (64.1)	514, 595, 757, 802

PCR=Polymerase chain reaction, *S. aureus=Staphylococcus aureus*

### Detection of coa gene variants by RFLP

**Figure-8:**
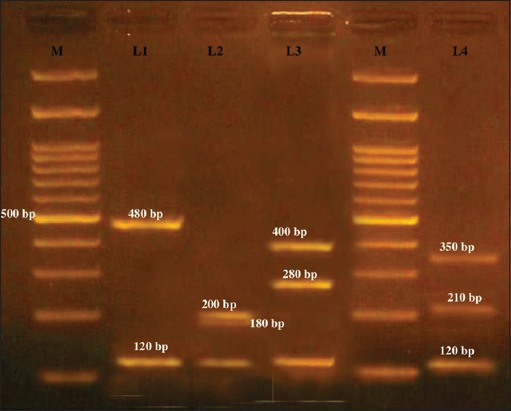
*Hae111* restriction enzyme digestion of *coa* variant polymerase chain reaction products. M - 100 bp ladder. L1 - *Hae111* digestion of approximately 600 bp *coa* gene variant into approximately 480 and 120 bp (P2). L2 - *Hae111* digestion of approximately 500 bp *coa* gene variant into approximately 200,180, and 120 bp (P1). L3 - *Hae111* digestion of approximately 800 bp *coa* gene variant into approximately 400, 280, and 120 bp (P4). L4 - *Hae111* digestion of approximately 750 bp *coa* gene variant into approximately 350, 210, and 120 bp (P3).

**Figure-9:**
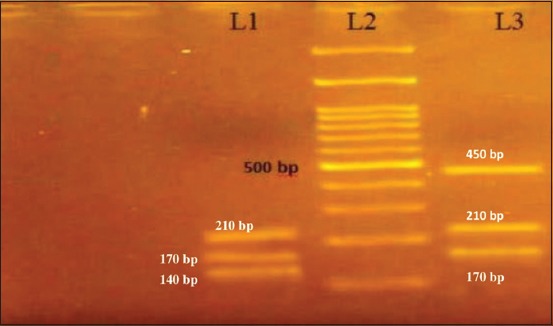
*Alu1* restriction enzyme digestion of 750 and 600 bp *coa* gene polymerase chain reaction products. L1 -*Alu1* digestion of approximately 600 bp coa gene variant into approximately 210, 170, and 140 bp (P2). L2 - 100 bp ladder. L3-*Alu1* digestion of approximately 750 bp *coa* gene variant into approximately 450, 210, and 170 bp (P3).

**Figure-10:**
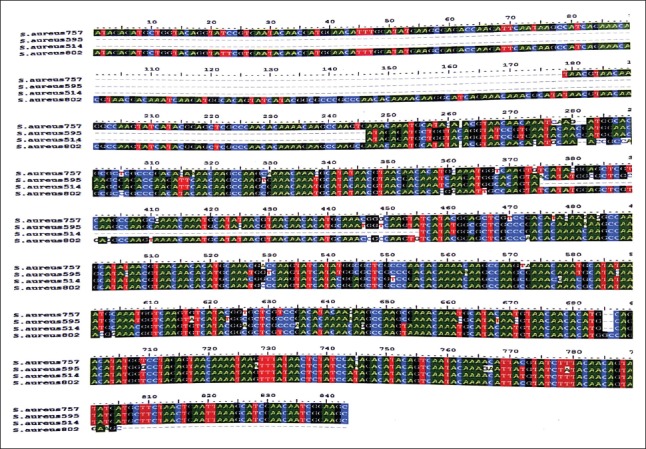
Sequence alignment using Bioedit software, to find maximum homology between the sequences of isolates, one each from four different restriction fragment length polymorphism patterns

**Figure-11:**
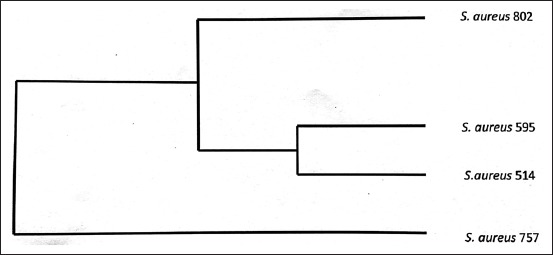
Dendrogram showing similarity among four *coa* variables having unique restriction patterns.

On *HaeIII* and *AluI* digestion of *coa* gene PCR product, four different RFLP patterns of *coa* gene variants were obtained (Figures-8 and 9, Table-3). Thus, four different RFLP patterns of *coa* gene variants were obtained in the present study. One strain each from different RFLP pattern was randomly selected and sent for sequencing (Xcelris Labs Ltd.). The sequences are yet to be submitted to NCBI, so accession number of the sequences are not generated yet. These sequences were aligned for maximum homology using Bioedit software (Figure-10). The similarity in the sequences was inferred with the help of sequence identity matrix as shown in Table-4 and a dendrogram was obtained (Figure-11). The most identical sequences with the value of 0.810 were found between isolate *S. aureus* 514, obtained from nasal cavity, and isolate *S. aureus* 595, obtained from mastitic milk. Thus, they are the most closely related, while as the isolates *S. aureus* 51*4* and *S. aureus* 802 are comparatively distant because of their least similarity with the value of 0.483 (Table-4).

**Table-3:** Restriction digestion pattern of *coa* variable isolates using restriction enzymes *HaeIII* and *AluI.*

*Coa* amplicon (approx. bp)	Number of isolates	Source	*HaeIII* digestion (bp approximately)	*AluI* digestion (bp approximately)	Pattern
500	2	Nasal swab	200, 180, 120		P1
600	15	Mastitic milk	480, 120	210, 170, 140	P2
750	4	Pus	350, 210, 120	450, 210, 170	P3
800	4	Mastitic milk	400, 280, 120	450, 260	P4

**Table-4:** Sequence identity matrix showing genetic homology among four *coa* variable isolates.

Seq‑>	*S. aureus* 757	*S. aureus* 595	*S. aureus* 514	*S. aureus* 802
*S. aureus* 757	ID	0.659	0.577	0.782
*S. aureus* 595	0.659	ID	0.810	0.539
*S. aureus* 514	0.577	0.810	ID	0.483
*S. aureus* 802	0.782	0.539	0.483	ID

S. aureus=Staphylococcus aureus

## Discussion

In our study, 39 samples comprising of 22 mastitic milk, 9 pus samples from skin wounds, and 8 nasal swab samples were found positive for *S. aureus*, which indicate their occurrence of 22.4%, 20.4%, and 16%, respectively in Military dairy farm, Belicharana, Jammu. The previous study showed that *S. aureus* accounts for 23% of etiological agents associated with clinical mastitis in 30 milk recording dairy herds [11]. Another report showed a prevalence of 33.9% of *S. aureus* in pus samples from cattle [12]. Similarly, 5.06% prevalence of *S. aureus* was reportedin the nasal cavity of cattle [13] in Iran. In another report in Norway, a prevalence of 13.9% of *S. aureus* in the nasal cavity of cattle was reported [14].

For molecular identification of *S. aureus* isolates, molecular targeting of species-specific *nuc* gene of *S. aureus* coding for the extracellular thermostable nuclease protein (TNase) of *S. aureus* was demonstrated by Louie *et al.*, and the same was utilized in the present study. PCR utilizing synthetic oligonucleotide primers of 21 and 24 bases has been used to amplify a segment of the *nuc* gene that is specific for *S. aureus* [15]. In our study, of 114 phenotypically identified Staphylococci, only 39 (34.2%) *S. aureus* isolates could be confirmed by *nuc* gene PCR. A study carried out on 68 *Staphylococcus* strains obtained from mastitic milk samples revealed 53 (78%) as positive for *nuc* gene [16]. Low detection of *S. aureus* inthe study may be attributed to non-utilization of other genotypic species-specific molecular procedures such as 23S rRNA-specific PCR, utilization of the crude method of DNA extraction, and lack of quantification that might increase the chances of false-negative results [17].

In our study, among all 39 isolates of *S. aureus* screened genotypically for *coa* gene, 25 strains (64.10%) amplified *coa* gene and are referred to as CoPS. All the 114 phenotypically identified Staphylococci cultures were subjected to tube coagulase test, which yielded positive coagulase test for 22 cultures. Three strains which were detected phenotypically by tube coagulase test as coagulase-negative were genotypically identified as coagulase positive. This emphasizes the use of molecular methods in the accurate detection of *coa* gene in *S. aureus*. A similar study carried out in 2015 found that 10 isolates, classified as coagulase-negative by tube coagulase test, were found to be positive with *coa* gene PCR [18].

PCR-RFLP is a rapid, reproducible, simple, and efficient method for typing *S. aureus* isolated from a clinical specimen. The variable region of *coa* gene is comprised of 81 bp tandem short sequence repeats. In our study, all the *coa*-positive isolates showed a single amplicon but of approximately four different sizes, 500 bp, 600 bp, 750 bp, and 800 bp. *S. aureus* isolates from mastitic milk source possessed *coa* amplicon of approximately 600 bp (15 isolates) and 800 bp (4 isolates). This finding is in agreement with a study carried out in 2007, which reported the presence of many variants of coagulase genotypes from regions of their study, but they indicated that only a few genotypes were predominant [19]. In a study carried out in 2015, all PCR products for *coa* gene were detected at 750 bp [18]. In our study, polymorphism of 4 *coa* gene products by RFLP using *HaeIII* and *AluI* restriction endonucleases was studied, and four RFLP patterns p1, p2, p3, and p4 were obtained. *HaeIII* showed better discrimination between the species than *AluI*. This is substantiated by a study carried out in 2014, which also concluded that *HaeIII* discriminatory power is better than *AluI* for typing *S. aureus* isolates [20]. One representative sample from each pattern was sequenced to obtain actual size of four *coa* types to be 514 bp, 595 bp, 757 bp, and 802 bp. Sequences of all the four patterns had unique restriction patterns. This was substantiated by unique restriction profile shown by these isolates on PCR-RFLP. Therefore, the study records the isolation of *S. aureus* with coagulase gene sequences having unique restriction patterns, not reported in earlier studies.

The sequences obtained were aligned for maximum homology between the sequences using Bioedit software [21], and similarity in the sequences was inferred with the help of sequence identity matrix. When the sequences from each of the distinct RFLP pattern were analyzed, it was found that the *S. aureus* isolate with a *coa* size of 514 bp, isolated from nasal cavity, had a highest similarity (81%) with the *S. aureus* isolate having *coa* size of 595 bp, isolated from mastitic milk. These results suggest that the possibility of *S. aureus* originating from nasal infections in the present study has evolved from mastitic milk strains and vice versa. When the genetic similarity of the other isolates was compared, it was found that a similarity of 48%, 53%, 57%, 65%, and 78% existed between the isolates *S. aureus* 802and *S. aureus* 514 (milk and nasal), *S. aureus* 595and *S. aureus* 802(both milk), *S. aureus* 757and *S. aureus* 514(pus from skin wound and nasal), *S. aureus* 602and *S. aureus* 757(milk and pus from skin wound),and *S. aureus* 800and *S. aureus* 757(milk and pus from skin wound),respectively. The genetic similarity among two rep­resentatives of *S. aureus* isolates from mastitic milk showed similarity only around 50% indicating evolutionary diversity in the strains causing mastitis in bovines.

## Conclusion

Thus, it is concluded from the present study that both *nuc* gene and 23S rRNA gene-specific PCR should be used simultaneously for the molecular identification of *S. aureus* as these molecular tests when used individually may not cover all the *S. aureus* isolates from the samples. Further,the present study was localized to only one farm and different RFLP patterns of coagulase gene were observed from different sampling sites, indicating that *S. aureus* coagulase types have site-specific predilection. Two *coa* patterns were observed in mastitic milk, indicating multiple origins of infection, with 595 bp *coa* genotype being predominant. 514 and 595 *coa* variants of *S. aureus* are genetically most related.

## Authors’ Contributions

AT and MAB designed the study. Laboratory work was done by FJ and assisted by AT and MAB. TAS helped in making understand the molecular part. GAB and MM prepared the manuscript and analyzed the data. All authors read and approved the final manuscript.
